# The study of structure and effects of two new proanthocyanidins from *Anogeissus pendula* leaves on rumen enzyme activities

**DOI:** 10.3389/fvets.2023.1163197

**Published:** 2023-04-20

**Authors:** Suman Lata, Pushpendra Koli, Sultan Singh, B. K. Bhadoria, Umesh Chand, Yonglin Ren

**Affiliations:** ^1^Plant Animal Relationship, Indian Council of Agricultural Research (ICAR)-Indian Grassland and Fodder Research Institute, Jhansi, India; ^2^School of Science, Health, Engineering and Education, Murdoch University, Murdoch, WA, Australia; ^3^Department of Microbiology, Central University of Punjab, Bathinda, India

**Keywords:** *Anogeissus pendula*, bioactivity score, drug score, epicatechin, gallocatechin, proanthocyanidins, ruminal enzymes

## Abstract

Two novel proanthocyanidins, (2R, 3R)-(+)-Gallocatechin-(4β → 8)4-(2R, 3R)-(+)-gallocatechin (compound 1) and 3-O-galloyl-(2S, 3S)-(–)-epicatechin-(4α → 8)-[3-O-galloyl-(2S, 3S)-(–)-epicatechin (4α → 8)]2-(2S, 3S)-(–)-epicatechin (compound 2), were structurally characterized from leaves of *Anogeissus pendula*. The structures were determined by ultraviolet spectroscopy (UV), proton nuclear magnetic resonance (^1^H NMR), ^13^C NMR, and heteronuclear multiple bond correlation. Molinspiration and Osiris property explorer applications were used to predict bioactivity and drug score. Drug scores of 0.08 and 0.05 were predicted for compounds 1 and 2, respectively. Predicted bioactivity scores were high. Due to their molecular weight, chemical structure, and conformation, the newly discovered proanthocyanidins possess an inclination to interact with proteins. Based on this premise, both compounds were subjected to *in vitro* testing against ruminal enzymes. They exhibited significant inhibition activities (*p* < 0.01) with a range of half maximal effective concentration (EC_50_) of 14.80–17.88 mg/mL of glutamic oxaloacetic transaminase in both protozoa and bacteria fractions. The ruminal glutamic pyruvic transaminase activity was significantly inhibited (*p* < 0.01) from EC_50_ 12.59–16.29 mg/mL, and R-cellulase inhibition was recorded with EC_50_ 18.20–21.98 mg/mL by compounds 1 and 2, respectively. Protease activity decreased with increasing incubation time and concentration of both compounds. The novel proanthocyanidins have potential roles in improving feed conversion ratios and in drug development.

## 1. Introduction

Polyphenolics are common secondary metabolites in plants that exhibit a wide range of sizes, structures, and functions. Although their chemical structures and functions are enigmatic, they generally interact with proteins and metal ions upon ingestion through multiple hydroxylation (1). Plant phenolics include various secondary metabolites, such as phenolic acids, flavonoids, coumarins, stilbenes, hydrolysable and condensed tannins, lignans, and lignins. These compounds exhibit antimicrobial, antioxidant, and anti-inflammatory properties, and their biological activity is determined by molecular structure (2). Of particular interest in livestock feed are proanthocyanidins (PA), a member of the phenolic compounds family and flavonoids subfamily. PAs may be foregut or hindgut fermenters and are defined by their ability to bind protein (3). Moderate concentrations of PA (2–4% dry matter) can exert beneficial effects on ruminants by slowing microbial digestion and enhancing the absorption of amino acids in the small intestine. However, there are reports of both positive and negative effects of PAs on animal diets, indicating their complex roles (4, 5). There are reports of how low tannin diets correspond with low digestibility, and high tannin diets with higher digestibility, an indication of the complex roles of PAs in the animal diet (6). Flavanols and their oligomers also interact with proteins. Interactions may be weak or strong; those formed are of low molecular size and remain in solution (7). The potential of using plant extracts from clove and mulberry leaves as feed additives in animal diets has been investigated, and it was found that they improved feed efficiency (8, 9). Hence, the structure vs. function relationship of polyphenolics defines whether the nutritional impact will be positive or negative. Understanding the structure–function relationship of polyphenolics is essential in determining their nutritional impact on animals (10).

This research focused on polyphenolics of a common small tree distributed throughout tropical Asia and Africa. The leaves of *Anogeissus pendula* Edgew (Combretaceae), known locally as Kardhai, are eaten by livestock, especially during lean periods, as sources of protein, energy, oil, fatty acids, and minerals (11, 12). In our previous studies, we assessed leaves of *A. pendula* for contents of crude protein (9.8–10.6%), neutral detergent fiber (46.4–58.6%), lignin (13.5–15.3%), and *in vitro* dry matter digestibility (25–38%) (13). Here, our research aimed to understand the phytochemical/structural properties of *A. pendula* phenolics (flavonoids or PAs) in relation to their effects on rumen enzyme activities (14–17). The use of natural compounds in animal feed has become an increasingly popular area of research, with the goal of improving animal health and productivity while minimizing negative environmental impacts. In our study, we investigated the potential of two novel compounds isolated from tree leaves *A. pendula* against ruminal enzymes to act as feed additives for ruminants, with promising results.

## 2. Materials and methods

### 2.1. Chemicals and reagents

Tannic acid, gallic acid, 2S, 3S(–)-epicatechin, 2R, 3R(+)-gallocatechin, 2S,3S(–)-epicatechin-3-O-gallate, and Sephadex LH-20 of analytical grade were purchased from Sigma, USA. All other reagents and solvents used were of analytical grade.

### 2.2. Isolation of proanthocyanidins

Leaves of *A. pendula* were harvested in the monsoon season from the Central Research Farm of ICAR-Indian Grassland and Fodder Research Institute, Jhansi, India. Harvested leaves were initially dried in shade and then placed in a hot air oven at 60°C until a constant dry weight was reached. The dried leaves were ground to a powder and passed through a 1-mm sieve. The powder was defatted using pure hexane (18). The defatted powder (4.5 kg) was placed in a Soxhlet extractor with pure ethanol. After Soxhlet extraction, the solvent was removed under vacuum in a rotatory evaporator at 40°C and suspended in 2 L of distilled water for 12 h. The remaining aqueous phase was washed with chloroform and ethyl acetate; then, the leftover extract (840 g) was chromatographed over a silica gel column (60–120 mesh). Gradient elution of the column with chloroform and methanol (60:40) yielded a yellow-colored solid, which was found to be a mixture monitored by thin-layer chromatography (TLC). This was purified on a pre-equilibrated Sephadex LH-20 column (30 × 2.5 cm) by eluting with H_2_O and methanol (10:1), which yielded a yellow crystalline compound containing two compounds that were resolved by preparative paper chromatography using 3MM Whatman paper and water as the irrigating solvent. The upper pink band was extracted with water and lyophilized to obtain compound 1, whereas compound 2 was a pale brown microcrystalline substance.

### 2.3. Characterization and structure determination

Melting points of the two compounds were determined using a Bock monoscope and were uncorrected. UV spectra were measured on a UNCAM UV/Vis spectrophotometer (Newington, USA). Mass spectra were determined on a Jeol mass spectrophotometer (Tokyo, Japan). ^1^H and ^13^C NMR spectra were obtained on Bruker DRX-300 spectrophotometer (Fallanden, Switzerland) with tetramethylsilane as an internal standard, and the heteronuclear multiple bond correlation (HMBC) was measured using a standard pulse sequence. High-performance liquid chromatography (HPLC) was carried out using a Shimadzu model LC-8A. The circular dichroism (CD) spectrum was done at the Department of Pharmacognosy, University of Mississippi, USA. TLC, column chromatography, and paper chromatography (PC) were performed on precoated Si GF^256^, Si gel (60–120 Mesh, Merck India), Sephadex LH-20 (Sigma, USA), and Whatman paper to characterize compounds 1 and 2.

### 2.4. Qualitative phytochemical investigation

Compounds 1 and 2 underwent complete acid hydrolysis to study anthocyanidin subunits through Shinoda, vanillin/HCL, and FeCl_3_ tests along with TLC and PC profiling (19). To determine monomeric units, compounds 1 and 2 were independently treated with phloroglucinol in the presence of 100 mL of 1% HCl in 50% aqueous methanol in a 250-mL round-bottom flask for 48 h. After drying of solvent, the product was diluted with H_2_O and extracted with ethyl acetate followed by evaporation. The dried product was dissolved in 80% methanol and subjected to quantitative analysis by 2D HPTLC (TLC plate cellulose; 20 × 20 cm), solvent of tertiary butanol: acetic acid: water at 3:1:1, and HPLC equipped with UV/VIS detector at 280 nm and RP ODS column (25cm × 4 mm, id) at ambient temperature with solvents of acetic acid (1%) (A) and methanol (B) at 1 mL/min.

### 2.5. *In silico* studies

*In silico* studies were performed using open-source software for virtual screening of the two novel compounds. Drug score value qualifies the overall potential of a compound as a drug candidate. OSIRIS property explorer was used to predict drug score by considering toxicity risks, partition coefficient between n-octanol and water (cLogP), solubility (logS), molecular weight (Mw), tropological polar surface area (TPSA), number of hydrogen acceptor and donor, number of rotatable bonds, and toxicity risks (20). Molinspiration is used to predict the bioactivity score of the isolated compound against regular receptors, such as GPCR ligand, ion channel modulators, kinase inhibitor, and nuclear receptor ligand (21).

### 2.6. *In vitro* ruminal enzyme activity

An adult sheep was selected for sampling from a small ruminant unit of the Plant Animal Relationship Division of IGFRI, Jhansi. Rumen liquor was collected before feeding. It is obtained through mouth using a perforated plastic tube with light suction in a 0.5-L capacity pre-warmed thermos (22). A ruminal cellulase extract was prepared from collected rumen liquor, and the effect of isolated compounds on its activities was estimated according to a described method (23). A protocol for determining the activity of the intracellular enzymes glutamic pyruvic transaminase (GPT) and glutamic oxaloacetic transaminase (GOT) was used (24) while obtaining from the bacterial and protozoal fractions of the rumen liquor and then separation of bacteria and protozoal rich enzyme extracts in 0.1 M phosphate buffer of pH 6.8 were carried out according to our published methods (17, 22, 25). To measure proteolytic enzyme activities, the concentration of protein in enzyme extracts was estimated according to Lowry (26). The proteolytic activity of isolated compounds was determined by estimating undigested protein from casein (27, 28).

### 2.7. Statistical analysis

For the statistical analysis, both Microsoft Excel 2016 and R (R-4.2.3) were used. To evaluate enzymatic activities, analysis of variance (ANOVA) was performed by using R, and significant differences in means were determined at *p* < 0.01 using *post-hoc* analysis with Tukey's test.

## 3. Results and discussion

### 3.1. Characterization of compounds 1 and 2

Compound 1: Pink amorphous substance, m.p. 280–82°C, UV(MeOH) λ_max_ 264 nm; FAB-MS [M+H]^+^ 1,827, C_90_H_74_O_42_; m/z; 1,718, 1,355, 1,216, 915, 911, 610, 305, and 167; CD spectral data, CD at 231.2 nm CD[medg] = 2.467, at 264.1 nm CD[medg] = 0.1784 and at 275.1 nm CD[medg] = 1.1191; ^1^H NMR, ^13^C NMR, and HMBC data are given in Table 1.

**Table 1:** ^1^H NMR, ^13^C NMR, and HMBC spectral data for compounds 1 and 2 in DMSO-d_6_ (δ, ppm, J/Hz)^*^.

**C atom**,		**Compound 1**		**Compound 2**	
**u, m, t**	**HMBC**	δ_H_	δ_C_	δ_H_	δ_C_
C-2 u, m		3.896 (5H, d, *J* = 8.4 Hz)	81.6	4.679 (3H, d, *J* = 4.2 Hz)	76.8
C-2 t		3.695 (1H, d, *J* = 8.1 Hz)	83.4	4.581 (1H, d, *J* = 3.6 Hz)	77.8
C-3 u, m		3.249 (5H, dd, *J* = 6.6, 7.5 Hz)	70.2, 73.1	5.646 (3H, dd, *J* = 4.5, 4.5 Hz)	74.5, 71.9
C-3 t		4.698 (1H, m)	61.5	3.929 (1H)	68.2
C-4 u, m		3.833 (5H, d, *J* = 8.7 Hz)	36.8	4.806 (3H, d, *J* = 6.3 Hz)	34.6
C-4 t		3.419 (2H, dd, *J* = 6.6, 7.8 Hz)	29.2	4.230 (2H, dd, *J* = 6.6, 6.3 Hz)	29.2
C-5			160.7		154.2
C-6 u, m	5,7 ^2^*J* & 8 ^3^*J*	6.185 (1H, s, H-6 u) 6.209 (5H, s, H-6 m, t)	89.6	6.179 (1H, s, C-6 u) 6.034 (3H, s, C-6 m)	96.0
C-6 t			89.6	6.034 (3H, s)	97.2
C-7			160.7		155.0
C-8 u		6.277 (1H, s)	102.7	6.467 (1H, s)	96.6
C-8 m, t			108.8		107.4
C-9			182.0		163.2
C-10			128.5		102.7
C-1′			137.8		130.8
C-2′	1′, 3′^2^*J* & 4′^3^*J*	7.312 (1H, s, H-2′ u) 6.745 (4H, s, H-2′ m) 6.438 (1H, s, H-2′ t)	121.4	7.555 (3H, s, H-2′ u, m) 6.998(1H, s, H-2′ t)	112.4
C-3′			145.7		145.5
C-4′			145.4		145.7
C-5′			145.7	7.927 (3H, *J* =8.7 Hz, H-5′ u, m) 6.949 (1H, *J* = 8.7 Hz, H-5′, t)	114.9
C-6′	1′, 5′^2^*J* & 4′^3^*J*	7.399 (1H, s, H-6′ u) 6.903 (4H, s, H-6′ m) 6.519 (1H, s, H-6′ t)	116.1	7.412 (3H, *J* = 7.2 Hz, H-6′, u, m) 6.769 (1H, *J* = 6.3 Hz, H-6′, t)	112.4
C-1″					122.7
C-2”				6.658 (4H, s)	110.7
C-3”					144.8
C-4”					139.3
C-5”					144.8
C-6”				6.570 (4H, s)	109.9
C-7”					166.8

u, upper unit; m, middle unit; t, terminal unit, ^*1^H, ^13^C, nuclear magnetic resonance (NMR) and 2D heteronuclear multiple bond correlation (HMBC) were determined on a Bruker DRX-300 spectrometer.

Compound 2: Pale brown microcrystalline substance, m.p. 270–72°C; UV(MeOH) λ_max_ 278 nm; FAB-MS [M+H]^+^ 1,611, C_81_H_62_O_36_; m/z; 1,458, 1,323, 1,305, 882, 730, 441, 303, 289, and 151; ^1^H NMR and ^13^C NMR data are given in Table 1.

**Figure 1:**
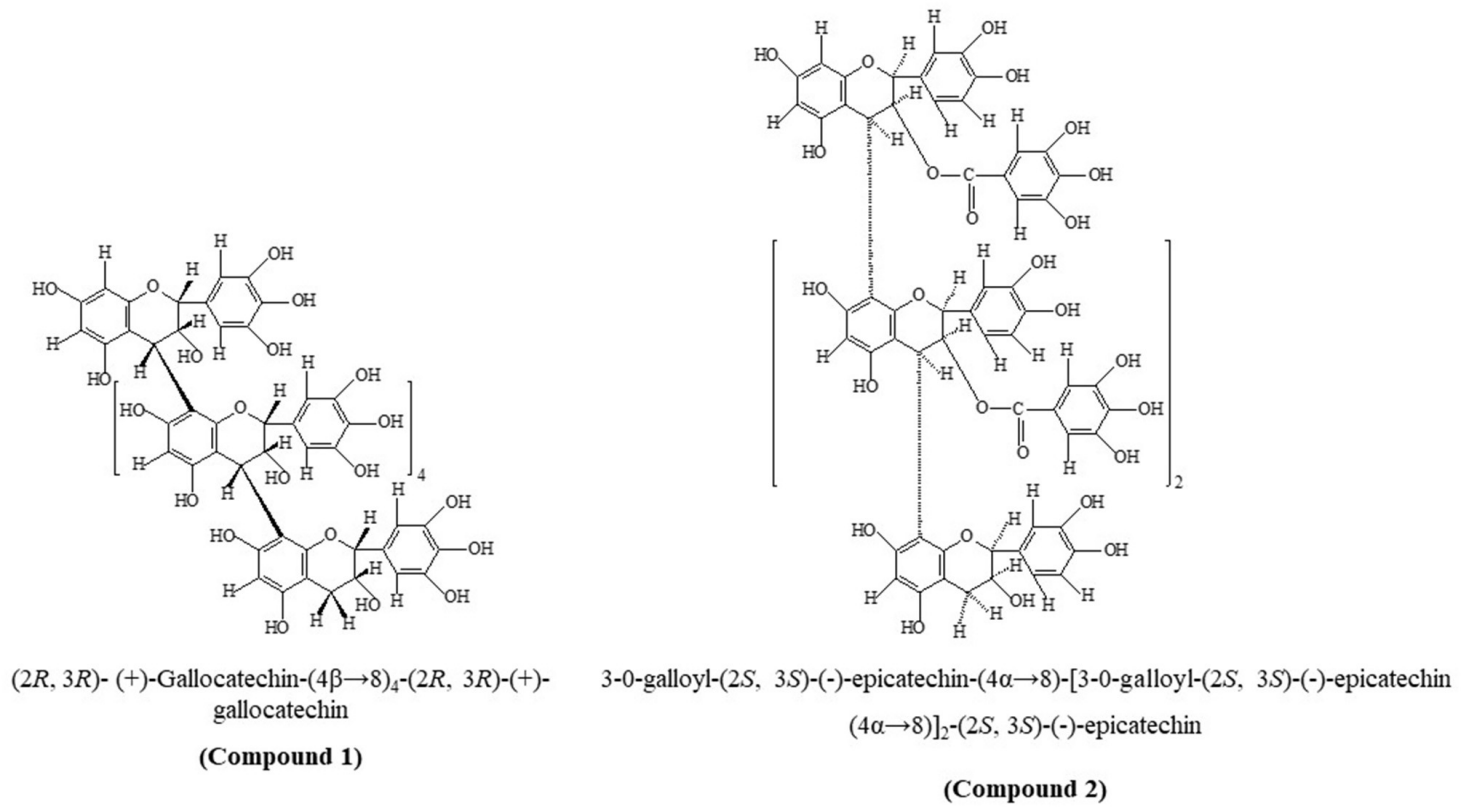
Chemical structures of compounds 1 and 2.

Compounds 1 and 2 (Figure 1) were obtained as pink and light brown microcrystalline substances, mp 280–82°C and 270–72°C, respectively, and were responsive to characteristic reactions of proanthocyanidin (29, 30). UV (MeOH, λ_max_, nm): 264 and 278 for compounds 1 and 2 further led us to infer the proanthocyanidin nature of the compounds (31). The furnished anthocyanidins after undergoing thorough acid hydrolysis (n-BuOH-HCl; 95:5) with compounds 1 and 2 identified as delphinidin (R_f_ 55) and cyanidin oligomeric procyanidin (R_f_ 45), respectively. The protonated fast atom bombardment mass spectrometry (FAB-MS) of compound 1 afforded a molecular ion peak (M+H) at 1,827, consisted of C_90_H_74_O_42_ furnishing molecular ion fragments due to retro-Diels-Alder (RDA) cleavage at m/z 1,718, 1,355, 1,216, 915, 911, 610, 305, and 167 confirmed the presence of (+)-gallocatechin in upper, middle, and terminal units (32) linked by C-C linkage (m/z 1,521 and 305). Furthermore, the molecular fragment at m/z 152 and 1,718 verified the structure as homogeneous oligomeric prodelphinidin. The protonated FAB-MS of compound 2 the M+H peak at m/z 1,611 with formula C_81_H_62_O_36_, furnishing molecular species due to consequence of RDA at m/z; 1,458, 1,323, 1,305, 882, 730, 441, 303, 289, and 151 confirmed the presence of galloyl moiety in upper and extender units (m/z 303 and 1,305), whereas the terminal unit was unsubstituted with galloyl unit (m/z 151 and 1,458) linked by C-C linkage (m/z 441, 882, and 1,323).

The polymeric nature of isolated compounds was verified by ^13^C NMR and ^1^H NMR (Table 1), and their physicochemical properties are depicted in Table 2. Chemical shift indicated for polyflavan-3-ol in both compounds; in addition, signals for galloyl moiety were also present in compound 2. Due to the complexity of structures, the spectra were studied as regions A and B (A: 30–90 ppm and B: 90–160 ppm) (33). In region A, out of 18 aliphatic carbons, six oxygenated methane carbons appeared at δ70.2, δ73.1, and δ61.5 for C-3 of upper (u), middle (m), and terminal (t) units, respectively. The up-field signal at δ81.6 and δ83.6 attributed to the C-2 of u, m, and t with 2,3-trans configuration and at δ36.8 and δ29.2, corresponding to the C-4 of u, m, and t units was indicative of 2,3-trans and 3,4-trans configuration (34). Of note was the observance of γ-gauche effect in ^13^C NMR for C-4 in ring-C at δ36.8 relative to δ81.6 for C-2 in the upper unit corroborated the 2,4-trans orientation in the prodelphinidin molecule (35). Region B of the spectrum displayed characteristic chemical shifts for 12 aromatic methine carbons at δ121.4 and δ116.1 for C-2′ and C-6′ of u, m, and t units, respectively. Hydroxy substituted carbons at δ145.7, δ145.4, and δ145.7 corresponded to C-3′, C-4′, and C-5′, respectively, of u, m, and t along with six quaternary carbons at δ137.8 of C-1′ of u, m, and t. The signals δ102.7 and δ108.8 were due to the C-8 carbon of u, m, and t units.

**Table 2:** Physicochemical properties of compounds 1 and 2 and standards used in this study.

**Compounds**	**IUPAC name**	**Solubility**	**Melting point (°C)**	**Molecular formula**
Compound 1	(2*R*, 3*R*)-(+)-Gallocatechin-(4β → 8)_4_-(2*R*, 3*R*)-(+)-gallocatechin	H_2_O	280–82°C	C_90_H_74_O_42_
Compound 2	3-O-Galloyl-(2*S*, 3*S*)-(–)-epicatechin-(4α → 8)-[3-O-galloyl-(2*S*, 3*S*)-(–)-epicatechin (4α → 8)]_2_-(2*S*, 3*S*)-(–)-epicatechin	H_2_O	270–72°C	C_81_H_62_O_36_
Tannic acid	[2,3-dihydroxy-5-[[(2R,3R,4S,5R,6S)-3,4,5,6-tetrakis[[3,4-dihydroxy-5-(3,4,5-trihydroxybenzoyl)oxybenzoyl]oxy]oxan-2-yl]methoxycarbonyl]phenyl] 3,4,5-trihydroxybenzoate	Alcohol, acetone, H_2_O	200°C	C_76_H_52_O_46_
Gallic acid	3,4,5-trihydroxybenzoic acid	H_2_O	258–265°C	C_7_H_6_O_5_
(–)-Epicatechin	(2*S*,3*S*)-2-(3,4-dihydroxyphenyl)-3,4-dihydro-2*H*-chromene-3,5,7-triol	H_2_O, Alcohol	235–237°C	C_15_H_14_O_6_
(+)-Gallocatechin	(2*R*,3*S*)-2-(3,4,5-trihydroxyphenyl)-3,4-dihydro-2*H*-chromene-3,5,7-triol	H_2_O	189–191°C	C_15_H_14_O_7_
(–)-Epicatechin-3-O-gallate	[(2*S*,3*S*)-2-(3,4-dihydroxyphenyl)-5,7-dihydroxy-3,4-dihydro-2*H*-chromen-3-yl] 3,4,5-trihydroxybenzoate	H_2_O	257–258°C	C_22_H_18_O_10_

Compound 2 exhibited 12 aliphatic carbons in region A in which four were oxygenated methine for C-3 of u, m, and t units that were represented by the chemical shift of δ74.5, δ71.9, and δ68.2, respectively. The up-field signal appeared at δ34.6 and δ29.2, corresponding to the C-4 of u, m, and t units with a 3,4-cis configuration (36). The up-field resonance of the heterocyclic ring carbon at δ76.8 and δ77.8 for C-2 of u, m, and t, respectively, indicated the 2,3-*cis* configuration. The absence of a γ-gauche effect in ^13^C NMR for C-4 (δ34.6) in ring-C relative to C-2 (δ76.8) in the upper unit strongly indicated 2,4-cis orientation with 4S configuration in the procyanidin molecule (36). In region B, the spectrum displayed chemical shifts for seventeen aromatic methine carbons at δ96.0 (C-6, u, m), δ97.2 (C-6, t), δ96.6 (C-8, u), δ112.4 (C-2′, u, m, t), δ114.9 (C-5′, u, m, t), and δ112.4 (C-6′, u, m, t), respectively, and hydroxyl substituted carbons at δ145.5 and δ145.7 corresponded for C-3′ and C-4′, respectively, of u, m, and t along with four quaternary carbons at δ130.8 of C-1′ of u, m, and t units. The resonance at δ107.4 was due to C-8 carbons of the m and t units. The A-ring carbons at C-7 and C-5 appeared at δ155.0 and δ154.2 in u, m, and t units. The chemical shifts at δ163.2 and δ102.7 were due to C-9 and C-10 of u, m, and t. The additional carbon signals at δ122.7 (C-1″), δ110.7 (C-2″), δ144.8 (C-3″), δ139.3 (C-4″) δ144.8 (C-5″), δ109.9 (C-6″), and δ166.8 (C-7″), respectively, confirmed the presence of galloyl moiety in the molecule.

The ^1^H NMR spectrum (DMSO-d6) of compound 1 showed the presence of singlets at δ6.185 (1H) and δ6.277 (1H) in aromatic region, which indicated a free proton each at C-6 and C-8, respectively, as confirmed by available HMBC relationships for C-4 → C-8 linkage between the upper and extension units (29). The appearance of doublets at δ3.896 (5H, *J* = 8.4 Hz) and δ3.833 (5H, *J* = 8.7 Hz) and a double doublet at δ3.249 (5H, *J* = 6.6, 7.5 Hz), forming AMX system (37) corresponding to C-2, C-4, and C-3 position, respectively, for upper and extension unit, exhibiting positive cotton effect at 231.2 nm (CD[medg] = 2.467) in the CD spectrum finally led to 4R configuration of protons with β linkage (38). The large coupling constant (*J* = 8.4 Hz and *J* = 8.7 Hz) for C-2 and C-3 was indicative of 2,3-*trans* orientation with β-linkage at C-4. Resonance forming AMX2 system by doublet at δ3.695 (1H, *J* = 8.1 Hz), multiplet at δ4.698 (1H), and double doublet at δ3.419 (2H, *J* = 6.6, 7.8 Hz) for C-2, C-3, and C-4 of terminal unit further corroborated of 2,3-*trans* configuration in terminal unit of molecule (39). The singlets at δ7.321 (1H), δ6.745 (4H), δ6.430 (1H) δ7.399 (1H), δ6.903 (4H), and δ6.519 (1H), respectively, are indicative of one proton at C-2′ and C-6′ of the B-ring of upper, extension, and terminal with ^2^J coupling with C-1′, C-3′ & C-1′, C-5′, and ^3^J coupling with C-4′ carbon, whereas the 1H NMR spectrum (DMSO-d6) of compound 2 demonstrated doublets at δ4.679 (3H, *J* = 4.2 Hz) and δ4.806 (3H, *J* = 6.3 Hz) and a double doublet at δ5.646 (3H, *J* = 4.5, 4.5 Hz), forming AMX system for C-2, C-4, and C-3 position of upper and middle units, respectively, suggested 2,3-*cis* orientation in upper and middle units as indicated by the low coupling constant (*J* = 4.2 and 4.5 Hz) for C-2 and C-3. The noteworthy up-field displacement of the C-3 proton suggested the presence of a methine proton attached with an oxygen-bearing carbon, indicative of galloyl moiety on the C-3 in upper and middle units (33). The presence of a doublet at δ4.581 (1H, *J* = 3.6 Hz), multiplet at δ3.929 (1H), and a double doublet at δ4.230 (2H, *J* = 6.6, 6.3 Hz), four protons, respectively, for terminal units inferred 2,3-*cis* configuration in the terminal unit. The chemical shifts appearing as singlets at δ6.179 (1H) and δ6.467 (1H) for C-6 and C-8, respectively, for the upper flavonoid moiety suggested C-4 → C-8 linkage with the middle unit. The presence of a singlet at δ6.034 (3H) indicated a C-6 proton of the m and t units. The free protons at C-2′ of the B-ring of u, m, and t units appeared as singlets at δ7.555 (3H) and at δ6.998 (1H), respectively. The protons for C-5′ and C-6′ of the B-ring of u, m, and t units were available as doublets at δ7.927 (3H, *J* = 8.7 Hz, H-5′ u, m), δ6.949 (1H, *J* = 8.7 Hz, H-5′, t), δ7.412 (3H, *J* = 7.2 Hz, H-6′, u, m), and δ6.769 (1H, *J* = 6.3 Hz, H-6′, t), respectively. The availability of protons at C-2′, C-5′, and C-6′ in the B-ring suggested the presence of an epicatechin unit in the u, m, and t units of the molecule. The chemical shifts as singlets at δ6.658 (4H) and δ6.570 (4H) for C-2″ and C-6″ suggested the presence of galloyl moieties in the molecule (33).

The acid treatment of isolated compound 1 with phloroglucinol yielded the (+)-gallocatechin and (+)-gallocatechin-4-phloroglucinol adduct, whereas compound 2 yielded flavanol (–)-epicatechin and 3-O-galloyl-(–)-epicatechin-4α-phloroglucinol, which were examined in HPLC. Compound 1 showed two peaks for (+)-gallocatechin (R_t_ = 22.54 min) with 2R:3R configuration and (2R:3R)-(+)-gallocatechin-4-phloroglucinol (R_t_ = 14.12 min), indicating the presence of (2R:3R)-(+)-gallocatechin in extension and terminal units in a molecule forming rare homogeneous oligomeric prodelphinidin (4, 40). In compound 2, we detected peaks for (–)-epicatechin (R_t_ = 28.32 min) with 2R:3R configuration and (2R:3R)-3-O-galloyl-(–)-epicatechin-4-phloroglucinol (R_t_ = 34.72 min), which suggested C-4 → C-8 inter-flavan linkage in procyanidin B type. This evidence was adequate to characterize 1 as hexameric (2R, 3R)-(+)-Gallocatechin-(4β → 8)4-(2R, 3R)-(+)-gallocatechin and 2 as B-3 type 3-O-Galloyl-(2S, 3S)-(–)-epicatechin-(4α → 8)-[3-O-galloyl-(2S, 3S)-(–)-epicatechin (4α → 8)]2-(2S, 3S)-(–)-epicatechin.

### 3.2. Drug and bioactivity scores

The bioactivity and drug scores of compounds 1 and 2 were predicted and compared with the standards tannic acid, gallic acid, 2S,3S(–)-epicatechin, 2R, 3R(+)-gallocatechin, and 2S,3S(–)-epicatechin-3-O-gallate (Tables 3A, B). The calculated drug score was 0.08 and 0.05 for compounds 1 and 2, respectively. The drug score combines druglikeness, cLogP (logarithm of partition coefficient), logS (logarithm of solubility), molecular weight, and toxicity risks in one value to judge a compound's overall potential as a drug (41). cLogP (octanol/water partition coefficient) is calculated through the methodology developed by Osiris property explorer (20), as a sum of fragment-based contributions and correction factors and used to predict the permeability of molecules across the cell membrane. Total polar surface area (TPSA) relates to hydrogen bonding potential of the molecule and is a predictor of drug transport properties, such as bioavailability, intestinal absorption, and blood–brain barrier penetration. Calculation of volume is based on group contributors. A number of rotatable bonds measure molecular flexibility, which is a descriptor of absorption and bioavailability of drugs (42).

**Table 3A:** ORISIS drug scores.

**Compounds**	**cLogP**	**TPSA**	**Druglikeness**	**H bond acceptor**	**H bond donor**	**Nb Stereocenters**	**Nb rotatable bonds**	**Drug-score**	**Solubility**
Compound 1	23.13	193.83	−7.55	21	18	3	3	0.08	−20.77
Compound 2	30.98	239.98	−9.18	26	19	7	7	0.05	−26.28
Tannic acid	5.53	777.98	1.60	46	25	5	31	0.31	−7.60
Gallic acid	0.11	97.99	0.12	5	4	0	1	0.27	−0.74
(–)-epicatechin	1.51	110.38	1.92	6	5	2	1	0.89	−1.76
(+)-Gallocatechin	1.96	240.99	2.39	13	11	5	3	0.35	−2.76
(–)-epicatechin-3-O-gallate	2.40	177.14	2.81	10	7	2	4	0.78	−2.46

**Table 3B:** Molinspiration bioactivity scores.

**Compounds**	**GPCR ligand**	**Ion channel modulator**	**Kinase inhibitor**	**Nuclear receptor ligand**	**Protease inhibitor**	**Enzyme inhibitor**
Compound 1	0.20	−0.14	−0.17	0.30	0.00	0.12
Tannic acid	−4.06	−4.07	−4.08	−4.08	−4.04	−4.05
Gallic acid	−0.77	−0.26	−0.88	0.52	−0.94	−0.17
(–)-epicatechin	0.41	0.14	0.09	0.60	0.26	0.47
(+)-Gallocatechin	0.15	−0.42	−0.18	0.07	0.15	0.04
(–)-epicatechin-3-O-gallate	0.17	0.02	0.05	0.34	0.13	0.25

The probability of bioactivity score of compound 1 toward a G protein-coupled receptor (GPCR) ligand, also called seven-transmembrane receptor or heptahelical receptor, nuclear receptor ligand, and enzyme inhibitor was 0.20, 0.30, and 0.12 (>0), respectively, which was shown to be active, and that of a kinase inhibitor, ion channel modulator, and protease inhibitor was −0.17, −0.14, and 0.00, respectively, which suggested the compound is moderately active (−5.0 to 0.0). For organic molecules, if the probability of bioactivity score is >0, then it is considered active. If the probability of bioactivity score range is −5.1 to 0.0, then it is considered moderately active, and if <-5.0, it is inactive (43, 44). The compounds showed results well within the active range, depicting a low risk of undesired behavior like mutagenicity or poor intestinal absorption, and thus indicated potential drug-like behavior. The results also confirmed the low risk of tumorigenic, irritant, and negative reproductive effects. The abovementioned software was unable to calculate the bioactivity scores for compound 2 due to its complexity.

### 3.3. Determination of effects on ruminal enzymes *in vitro*

**Figure 2:**
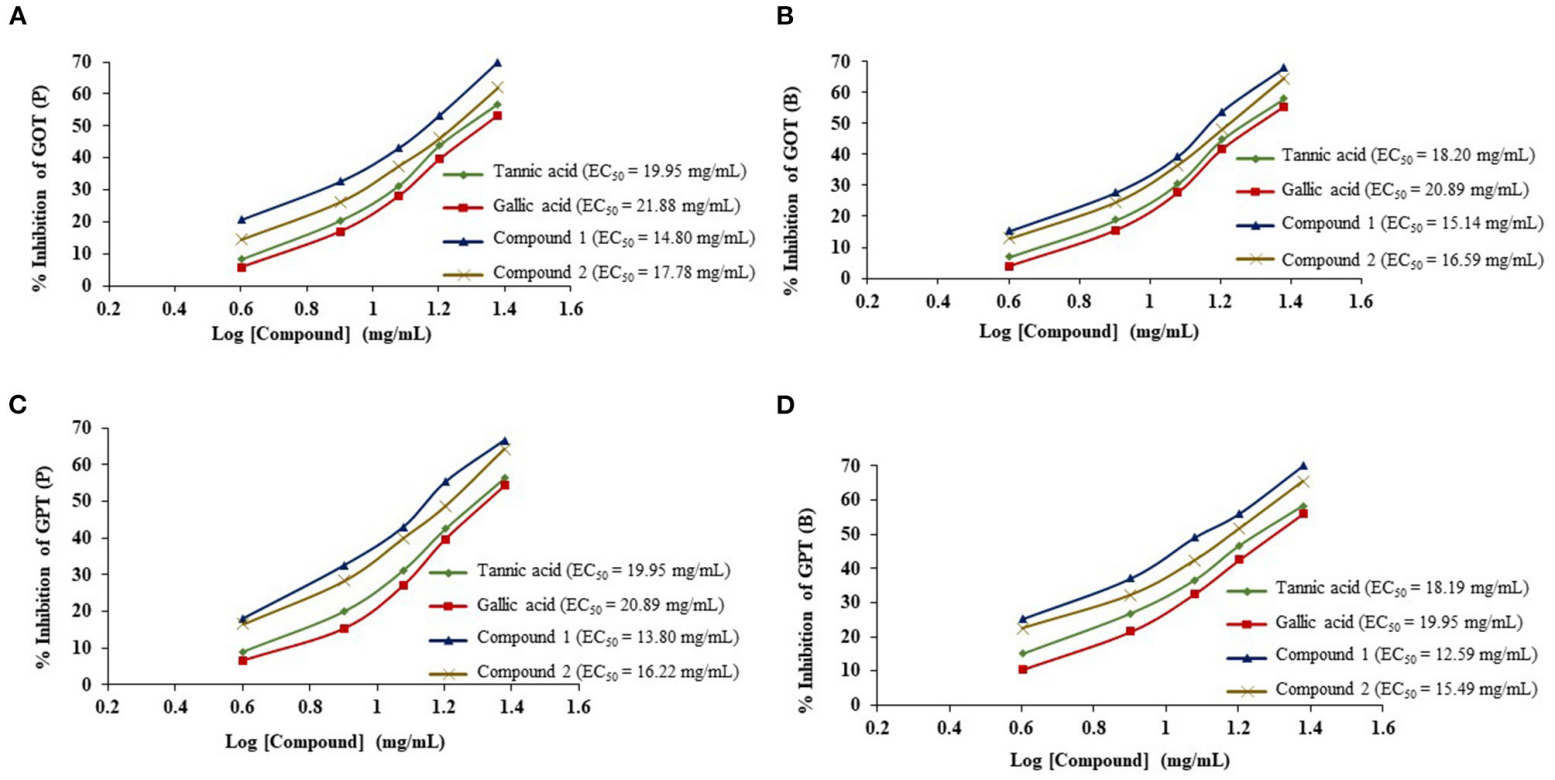
Effects of compounds 1 and 2 compared with tannic acid and galic acid on inhibition activity (EC_50_) of ruminal glutamic oxaloacetic transaminase (R-GOT) (**A**: protozoa fraction, **B**: bacterial fraction) and ruminal glutamic pyruvic transaminase (R-GPT) (**C**: protozoa fraction, **D**: bacterial fraction).

**Figure 3:**
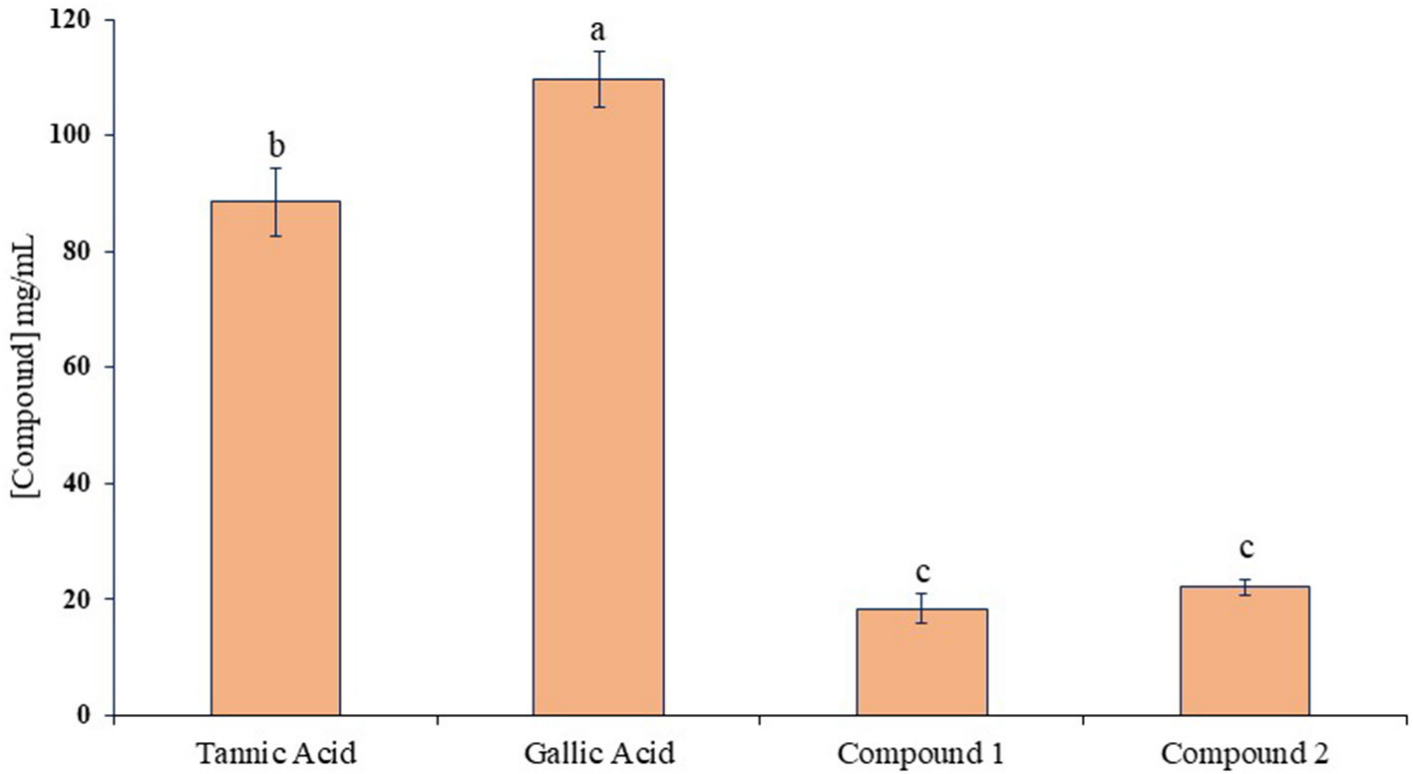
Inhibition activity (EC_50_) against ruminal cellulase for compounds 1 and 2 compared with tannic acid and gallic acid. Different letters above the bars indicate significant differences between the treatments.

The ruminal glutamic oxaloacetic transaminase (R-GOT), glutamic pyruvic transaminase (R–GPT), and cellulase activities are illustrated in Figures 2A, B. Compounds 1 and 2 significantly (*p* < 0.01) inhibited the activities of R-GOT (P) with EC_50_ 14.79 and 17.78 mg/mL and R-GOT (B) with EC_50_ 15.14 and 16.60 mg/mL, respectively. The R-GPT activity was also inhibited significantly (*p* < 0.01) in the presence of compounds 1 and 2 (Figures 2C, D). The comparison of inhibition in protozoal and bacterial fraction envisaged nearly equal for EC_50_ (mg/mL) activity as 13.80 and 12.60, respectively, for compound 1 and 16.23 and 15.49 for compound 2. The effect on cellulase activity of compound 1 showed strong inhibition of EC_50_ 18.197 mg/mL compared with compound 2 with an EC_50_ 21.878 mg/mL (Figure 3). This reveals a strong affinity of compound 1 to bind cellulase enzyme in less quantity, and consequently, this might have effects on fiber digestibility. Tannic acid and gallic acid exhibited significantly (*p* < 0.01) higher reduction in both GPT and GOT than did compounds 1 and 2. In the case of cellulase enzyme activity, tannic acid was more effective than gallic acid as the effective concentration to inhibit 50% activity (EC_50_) was 89.13 and 109.65 mg/mL for gallic acid and tannic acid, respectively. Both compounds 1 and 2 significantly (*p* < 0.01) inhibited cellulase activity compared to both standards.

This inhibition effect of phenolic compounds could be a result of their antimicrobial nature and the release of other metabolites during the fermentation process. Similar observations were recorded in cow rumen kinetics (14) from phenolic extracts of *Ficus* species (17) and methanolic tree leaves extracts of *A. pendula* (25). The released or break-down products of phenolic compounds from the plant extracts can be turned into new antioxidants (45) and that could reduce ruminal enzymatic activities. The inhibitory effects of legume-extracted phenolics on cellulose digestion (46) help to support our findings on the reduction of ruminal cellulase activity. The effects of simple phenolic acids also showed a significant decrease in the activities of rumen enzymes *in vitro* (47).

**Figure 4:**
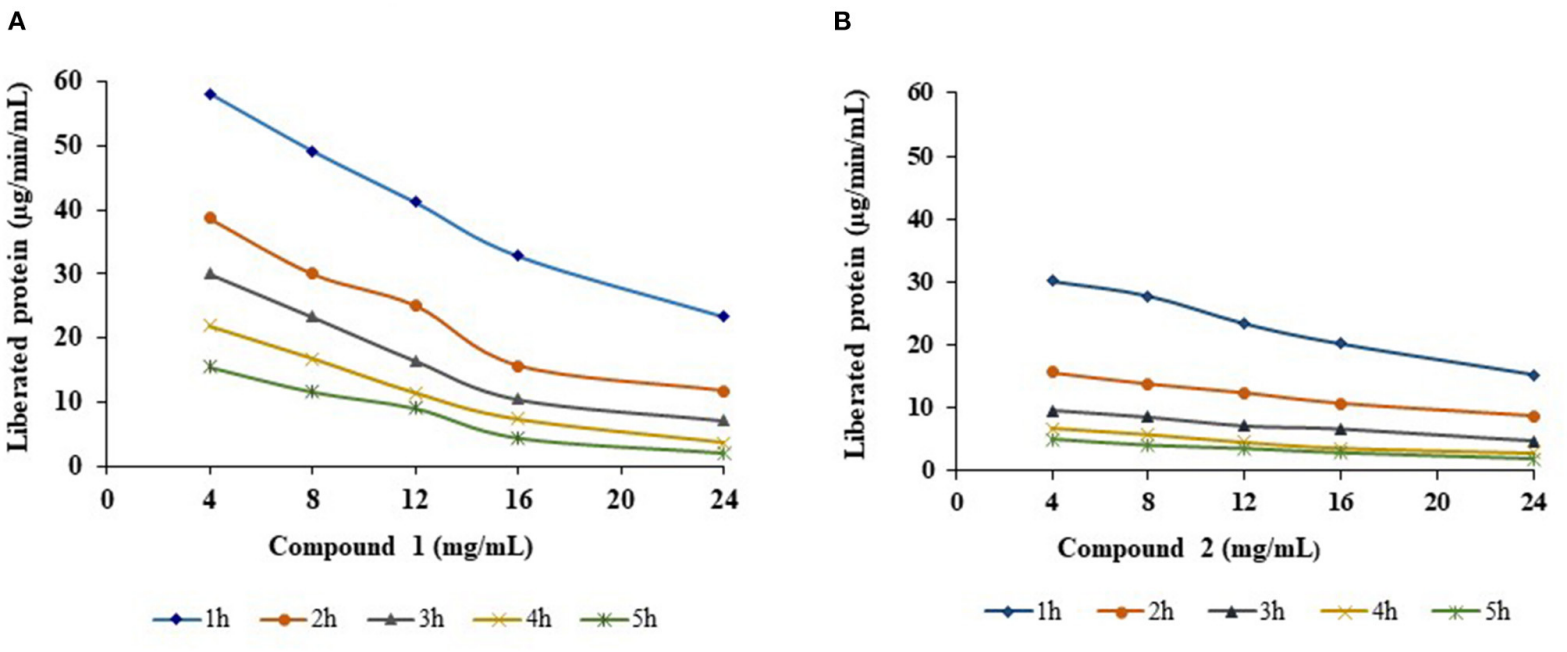
Effect of compounds 1 **(A)** and 2 **(B)** on R-protease with different concentrations and different incubation times.

R-protease activity of both compounds was significantly (*p* < 0.01) decreased proportional to increasing concentration (4, 8, 12, 16, and 24 mg/mL) and duration of incubation time (1, 2, 3, 4, and 5 h) (Figure 4). Compound 1 proteolysis decreased linearly with increasing time and increasing quantity. The amount of liberated protein (μg/min/mL) was 2.02 at 24 mg/mL at 5 h, whereas it was 58 at 4 mg/mL in the 1st h. A similar trend was observed with compound 2; a decline in protease activity was reported between 7–34% and 21–50% with each increasing concentration and increasing incubation time. At the highest concentration (24 mg/mL), the amount of liberated protein was lowered to 1.87 μg/min/mL.

The trend for reduction in the concentration of liberated rumen protein by the addition of compounds 1 and 2 with increasing concentrations and incubation times was also observed by others with condensed tannins extracted from *Lotus pedunculatus* (48), proanthocyanidins obtained from *Ficus* species (17), and tannin-rich forage leaves (49, 50). This could be due to the steric interference at interaction sites of protease and receptors. The basic route of proteolysis inhibition by phenolic compounds is based on interference with the interaction of enzyme substrates (51). In addition to this, the different types of phenolic structures and the nature of protein vary by the degree of binding. It is believed that the presence of proanthocyanidins/phenolics increases protein flow from rumen to intestine, where it is directly available to the animal (52). Thus, proanthocyanidins can have beneficial effects if they bind protein (50) or detrimental effects if they lower ruminal digestion without binding the protein, particularly for hemicellulose (53). The isolated compounds from leaves of *A. pendula* can be potentially used as a natural and sustainable additive in animal feed to improve nutrition and minimize health risks and environmental pollution. Novel animal feed formulations can be developed that incorporate these compounds, and their testing in controlled animal feeding trials to evaluate their effects on animal growth, health, and wellbeing. The compounds could also be tested for their ability to reduce environmental pollution by reducing the excretion of harmful compounds in animal waste.

## 4. Conclusion

The molecular structures of two novel polyphenolic compounds isolated from *A. pendula* leaves were elucidated, and their activities were tested. Both compounds inhibited the activity of all ruminal enzymes tested. These compounds can be developed into dietary supplements or functional food for animals to enhance the utilization of nutrients. The mode of action of polyphenolics and proanthocyanidins in the gut is not fully understood, so relating molecular structure to the mechanisms and actions by which different proanthocyanidins elicit depression in intake and digestibility in bovines is required. The discovery of these novel compounds expands our understanding of diverse and complex roles of proanthocyanidins in the animal diet and highlights the potential for further investigation into the molecular–function relationship of these compounds. Further research is needed to evaluate the safety and efficacy of these compounds *in vivo*, as well as to optimize their production and extraction from natural sources.

## Data availability statement

The original contributions presented in the study are included in the article/supplementary material, further inquiries can be directed to the corresponding authors.

## Author contributions

SL, PK, SS, BB, and YR: conceptualization. SL, PK, SS, UC, and YR: writing–original draft preparation and writing—review and editing. SS, BB, and YR: supervision. All authors have read and agreed to the published version of the manuscript. All authors contributed to the article and approved the submitted version.
